# A Novel Calibration Method of Articulated Laser Sensor for Trans-Scale 3D Measurement

**DOI:** 10.3390/s19051083

**Published:** 2019-03-03

**Authors:** Jiehu Kang, Bin Wu, Xiaodeng Duan, Ting Xue

**Affiliations:** 1 State Key Laboratory of Precision Measuring Technology and Instruments, Tianjin University, Tianjin 300072, China; zhongdian313@163.com (J.K.); duanxiaodeng@tju.edu.cn (X.D.); 2 School of Electrical and Information Engineering, Tianjin University, Tianjin 300072, China

**Keywords:** articulated laser sensor, calibration, perspective projection model, image processing, laser beam

## Abstract

The articulated laser sensor is a new kind of trans-scale and non-contact measurement instrument in regular-size space and industrial applications. These sensors overcome many deficiencies and application limitations of traditional measurement methods. The articulated laser sensor consists of two articulated laser sensing modules, and each module is made up of two rotary tables and one collimated laser. The three axes represent a non-orthogonal shaft architecture. The calibration method of system parameters for traditional instruments is no longer suitable. A novel high-accuracy calibration method of an articulated laser sensor for trans-scale 3D measurement is proposed. Based on perspective projection models and image processing techniques, the calibration method of the laser beam is the key innovative aspect of this study and is introduced in detail. The experimental results show that a maximum distance error of 0.05 mm was detected with the articulated laser sensor. We demonstrate that the proposed high-accuracy calibration method is feasible and effective, particularly for the calibration of laser beams.

## 1. Introduction

Nowadays, accompanied with the rapid development of manufacturing, 3D measurement has been widely applied in the domains of complicated surfaces measurement, dynamical scan, and reverse engineering [1,2,3,4]. The traditional 3D measurement instruments include the laser tracker [5,6], total station [7], and theodolite [8]. Their measurement accuracies rely heavily on the structure orthogonality, which increases their expenditures. Structured light 3D scanners and laser vision sensors are widely applied techniques in 3D measurement space [9]. However, the cameras of vison sensors are difficult to apply to trans-scale space measurement, because their limited focus leads to a dead measurement distance beyond which objects cannot clearly be seen.

For the trans-scale and non-contact measurement in regular-size space and industrial applications, there are many deficiencies and application limitations for traditional measurement methods. In reference to the three axes architecture of traditional instruments, an articulated laser sensor combined with adaptive focusing technology of laser alignment is proposed. However, the calibration method of system parameters for traditional instruments is no longer suitable for articulated laser sensors. Wu et al. proposed a non-orthogonal shaft laser theodolite (N-theodolite) [10]. The intrinsic parameters were obtained by minimum-zone circle fitting and linear fitting. Moreover, by aiming the targets at a scale bar placed at several different positions, the extrinsic parameters can be calibrated. However, this calibration method is suitable only for large-size measurements and low accuracy. The articulated laser sensor employs N-theodolite’s non-orthogonal architecture. It is essential to study the method of calibrating the spatial position of the laser beam, which represents the visual measuring axis. The spatial position consists of the direction vector of the laser beam and a fixed point on the laser beam.

Bi et al. mounted a laser displacement sensor on the *Z*-axis of a coordinate measuring machine (CMM) to build up an optical coordinate measuring system, and proposed a method based on a standard sphere to calibrate the laser beam direction. This method required length information from laser displacement sensor [11]. Sun et al. presented a vision measurement model of the laser displacement sensor, and then achieved the calibration with a planar target mounted on a 2D moving platform. However, during the calibration, it was difficult to realize that the planar target was perpendicular to the fixed plane of the moving platform [12]. Xie et al. established a multi-probe measurement system, and proposed a technique called the “coplanar calibration method” to calibrate the extrinsic parameters of the structured-light sensor. This method is not suitable to determine the initial spatial position of laser beam [13]. Yang et al. proposed a kind of inner diameter measuring device by increasing the number of laser displacement sensors. The method can calibrate the direction of three laser beams simultaneously [14]. The issue is that the methods of laser beam calibration can only obtain the direction vector of the laser beam [15,16,17]. Moreover, the spatial position of the laser beam cannot be received through the direction vector.

To achieve the calibration requirements of the articulated laser sensor, a novel calibration method is proposed in this paper. The key innovative aspect of this paper is the proposed method to calibrate the spatial position of laser beam.

The remainder of this paper is organized as follows. In Section 2, the principle of the articulated laser sensor is introduced. In Section 3, the calibration method of the articulated laser sensor is presented. Particularly, the calibration method of the laser beam is introduced in detail. In Section 4, the image processing of the laser spot is presented. In Section 5, the actual measurement experiments are performed. Those experimental data validate that the proposed method is effective. The paper ends with some concluding remarks in Section 6.

## 2. Principle of Articulated Laser Sensor

### 2.1. System Construction

**Figure 1:**
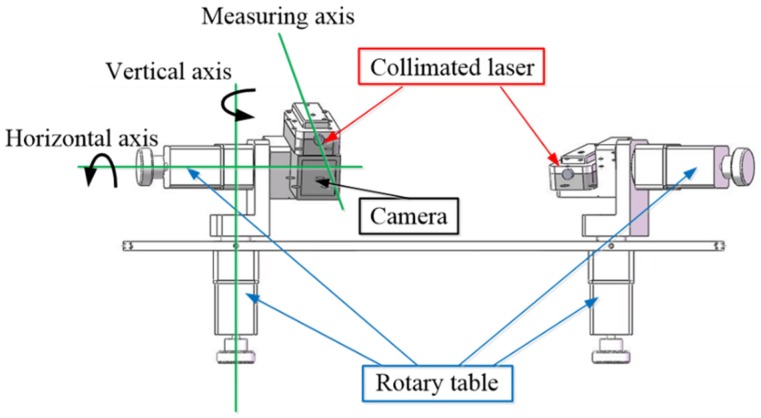
Structural diagram of articulated laser sensor.

The trans-scale 3D coordinate measurement system mainly consists of two articulated laser sensing modules, as shown in Figure 1. Each module is made up of two one-dimensional rotary tables and one collimated laser conveniently. As with traditional orthogonal measurement instruments, there are three axes in the articulated laser sensing module. However, the three axes have no strict requirements for the orthogonality and intersection conditions. The three axes are bifacial straights, and the angle between any two axes is not 90°. The rotating axes of the rotary tables of the articulated laser sensing module are called the “vertical axis” and “horizontal axis”, respectively, and the location of collimated laser beam is called the “measuring axis”.

### 2.2. Measurement Principle

**Figure 2:**
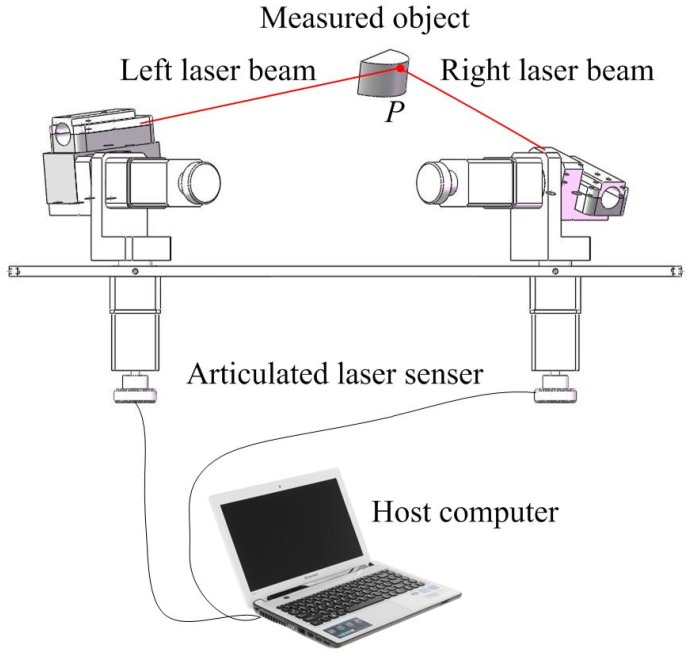
Structural diagram of the measurement system.

Similar to determining 3D coordinates utilizing traditional forward intersection measurement instruments, the measurement operation of the articulated laser sensor is based on the intersection of two laser beams in the measured object. With the help of a high-resolution digital CCD camera, the intersection can be achieved accurately. During measurement, the coincidence of two laser beams on the measured object denotes the intersection of the visualized measuring axes. As shown in Figure 2, when the left laser beam coincides with the right laser beam on a point of the measured object, the 3D coordinate of the point can be calculated based on the rotation angles provided by the rotary tables of two articulated laser sensing modules and the mathematical measurement model. The mathematical measurement model is established based on the perspective projection model and quaternion kinetic model.

## 3. Calibration Principle

**Figure 3:**
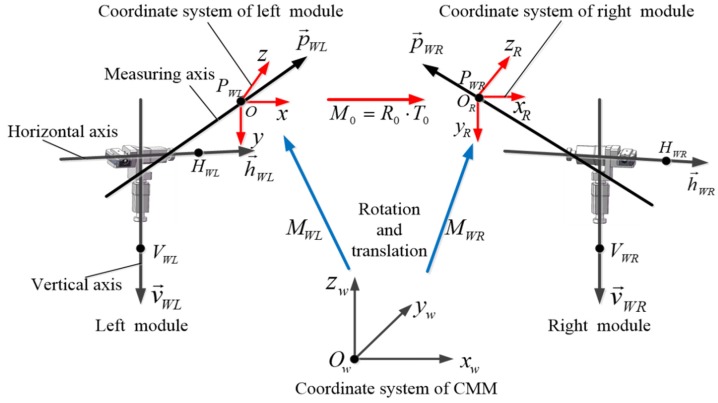
Schematic diagram of parameters calibration.

The system parameters calibration of the articulated laser sensor is necessary to achieve high-accuracy measurement, as the sensor measurement accuracy is greatly affected by the calibration method [18,19]. The system parameters consist of intrinsic and extrinsic parameters. For determining 3D coordinates, it is necessary to obtain the related positions of the three axes of the articulated laser sensing module, which are called the intrinsic parameters. The extrinsic parameters denote the relationship between the left module of the articulated laser sensor and the right module and include a rotation matrix $R_{0}$ and a translation vector $T_{0}$. The three axes of every articulated laser sensing module can be abstracted as three lines in 3D space, as shown in Figure 3. The system parameters of the articulated laser sensor and their physical meanings are listed in Table 1.

### 3.1. Calibration of the Vertical and Horizontal Axes

To make precise measurements, it is necessary to calibrate the parameters with a high-accuracy measurement instrument. A CMM is employed to calibrate these intrinsic and extrinsic parameters of the articulated laser sensor in the laboratory. The accuracy of the CMM is 2.1 μm + 2.8*l*/1000 μm, and *l* is the measurement distance. Two porcelain beads with diameters of 7.935 mm and a machining accuracy of 0.25 μm are pasted onto the two articulated laser sensing modules, respectively. Then, the two articulated laser sensing modules are rotated vertically and horizontally every ten degrees, and the centers of the porcelain bead are measured in each position. Thirty-six measured data points are obtained and these data are measured from a complete ellipse. The least square method is utilized to optimize the parameters. The direction vectors and fixed points of three axes are needed for calibration. The parameters of vertical and horizontal axes are obtained as follows:

(1) Based on least squares methods, a plane is fitted utilizing the centers of the porcelain beads, which are measured by CMM.
(2) The distances from the measured points to the fitted plane are calculated. If the distance is more than the threshold, these points are eliminated and another plane is fitted again.
(3) The normal vector of fitted plane is recorded as the direction vector of the rotation axis.
(4) The remaining points are projected onto the fitted plane.
(5) Based on least squares methods, an ellipse is fitted utilizing the projected points.
(6) The center of fitted ellipse is recorded as the fixed point of rotation axis.

In the measurement space, a plane can be expressed as [20]

(1) f(Θ,P)=Ax+By+Cz+D=0

where the vector Θ=[A,B,C,D] contains the plane parameters and P=(x,y,z) represents a point on the plane.

The four parameters of Θ=[A,B,C,D] are redundant. Equation (1) can be simplified as

(2) ax+by+cz=1

where $a=-\frac{A}{D},\;b=-\frac{B}{D},\;c=-\frac{C}{D}$.

Equation (2) takes the form of the matrix equation

(3) X∗L=Y

where X=(x,y,z) is obtained from measured data, $L={\left[a,b,c\right]}^{T}$ denotes unknown parameter, and $Y={\left[1,1,1,\ldots ,1\right]}^{T}$.

The number of measured data is more than the unknown parameter. Equation (3) is overdetermined and solved by least squares methods. The normal vector of the fitted plane is recorded as

(4) $$\vec{n}=(a,b,c)$$

The unit normal vector is defined as the direction vector of the rotation axis. The distances from the measured points to the fitted plane are expressed as

(5) $$d=\frac{ax+by+cz-1}{\sqrt{a^{2}+b^{2}+c^{2}}}$$

The point is eliminated if |d|>0.005 mm. The remaining points are projected onto the fitted plane, and the matrix equation is expressed as

(6) $$\left[\begin{array}{l} x^{\prime }\\ y^{\prime }\\ z^{\prime } \end{array}\right]=\left[\begin{array}{l} x\\ y\\ z \end{array}\right]-d*\vec{n}$$

These projected points are used for fitting the ellipse, and the center is defined as the fixed point of the rotation axis. An ellipse can be expressed as [21]

(7) $$f(\Theta^{\prime },p)=A^{\prime }{x^{\prime }}^{2}+B^{\prime }x^{\prime }y^{\prime }+C^{\prime }{y^{\prime }}^{2}+D^{\prime }x^{\prime }+E^{\prime }y^{\prime }+F^{\prime }=0$$

where the vector $\Theta^{\prime }=[A^{\prime },B^{\prime },C^{\prime },D^{\prime },E^{\prime },F^{\prime }]$ contains the ellipse parameters and $p=(x^{\prime },y^{\prime })$ is a point on the ellipse.

The six parameters of $\Theta^{\prime }=[A^{\prime },B^{\prime },C^{\prime },D^{\prime },E^{\prime },F^{\prime }]$ are redundant. Equation (7) can be simplified as

(8) $$a^{\prime }{x^{\prime }}^{2}+b^{\prime }x^{\prime }y^{\prime }+c^{\prime }{y^{\prime }}^{2}+d^{\prime }x^{\prime }+e^{\prime }y^{\prime }=1$$

where $a^{\prime }=-\frac{A^{\prime }}{F^{\prime }},\;b^{\prime }=-\frac{B^{\prime }}{F^{\prime }},\;c^{\prime }=-\frac{C^{\prime }}{F^{\prime }},\;d^{\prime }=-\frac{D^{\prime }}{F^{\prime }},\;e^{\prime }=-\frac{E^{\prime }}{F^{\prime }}$.

Equation (8) takes the form of a matrix equation:

(9) $$X^{\prime }*L^{\prime }=Y^{\prime }$$

where $X^{\prime }=({x^{\prime }}^{2},x^{\prime }y^{\prime },{y^{\prime }}^{2},x^{\prime },y^{\prime })$ is obtained from projection data, $L^{\prime }={\left[a^{\prime },b^{\prime },c^{\prime },d^{\prime },e^{\prime }\right]}^{T}$ denotes unknown parameter, and $Y^{\prime }={\left[1,1,1,\ldots ,1\right]}^{T}$. The center of ellipse is expressed as

(10) $$\left\{ \begin{array}{l} x_{c}=\frac{b^{\prime }e^{\prime }-2c^{\prime }d^{\prime }}{4a^{\prime }c^{\prime }-{b^{\prime }}^{2}}\\ y_{c}=\frac{b^{\prime }d^{\prime }-2a^{\prime }e^{\prime }}{4a^{\prime }c^{\prime }-{b^{\prime }}^{2}} \end{array}\right.$$

In summary, the direction vector of the rotation axis is expressed as Equation (4), and the fixed point is expressed as Equation (10).

### 3.2. Calibration of the Laser Beam

In 3D space, the laser beam can be abstracted as a spatial line. In the world coordinate system, the equation of the laser beam can be expressed as

(11) $$\frac{x_{w}-x_{1}}{x_{2}-x_{1}}=\frac{y_{w}-y_{1}}{y_{2}-y_{1}}=\frac{z_{w}-z_{1}}{z_{2}-z_{1}}$$

where $\left(x_{1},y_{1},z_{1}\right)$ and $\left(x_{2},y_{2},z_{2}\right)$ are the coordinates of the laser spots. Therefore, the key aspect is to obtain the coordinates of the laser spots in the world coordinate system.

**Figure 4:**
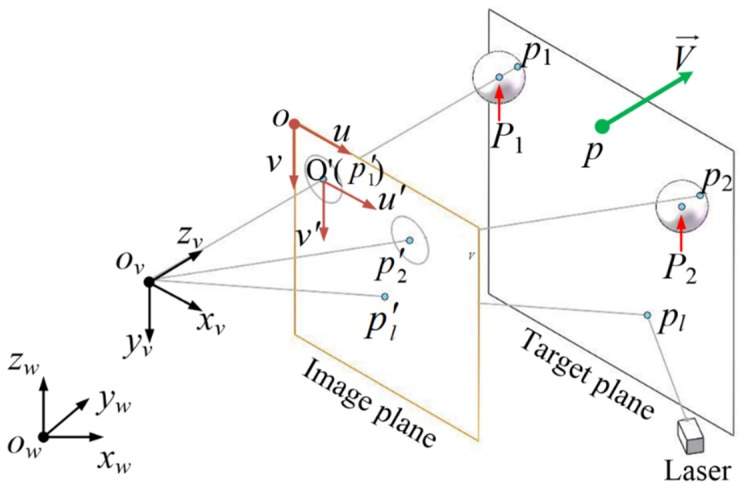
The calibration diagram of the laser beam.

The calibration principle diagram is shown in Figure 4. There are two parallel planes which are called the “image plane” and the “target plane”, respectively. The method used to ensure that the two planes are parallel is introduced in Section 4. Two porcelain beads are pasted to the target plane. The centers of the two porcelain beads are defined as $P_{1}$ and $P_{2}$. The projection of $P_{1}$ and $P_{2}$ onto the target plane are defined as $p_{1}$ and $p_{2}$. The projection of $p_{1}$ and $p_{2}$ onto the image plane are defined as ${p^{\prime }}_{1}$ and ${p^{\prime }}_{2}$. The laser spot on the target plane is defined as $p_{l}$, and the projection onto the image plane is defined as ${p^{\prime }}_{l}$.

Based on the principle of calibration, four coordinate systems are established:

(1) The world coordinate system is defined as $o_{w}-x_{w}y_{w}z_{w}$. The CMM’s measurement coordinate system is regarded as world coordinate system.
(2) The viewpoint coordinate system is defined as $o_{v}-x_{v}y_{v}z_{v}$. The $z_{v}$ axis is perpendicular to the target plane, and the $x_{w}$ and $y_{w}$ coordinates of origin $o_{v}$ are equal to the $x_{w}$ and $y_{w}$ coordinates of $p_{1}$.
(3) The actual coordinate system of pixels on CCD is defined as o−uv.
(4) The image plane coordinate system is defined as $o^{\prime }-u^{\prime }v^{\prime }$. The $u^{\prime }$ axis is parallel to $x_{v}$ axis, and the u and v coordinates of origin $o^{\prime }$ are equal to the u and v coordinates of ${p^{\prime }}_{1}$.

The equation of the target plane in the world coordinate system is expressed as

(12) $$l(x_{w}-x_{w}^{\prime })+m(y_{w}-y_{w}^{\prime })+n(z_{w}-z_{w}^{\prime })=0$$

where $\vec{V}=\left(l,m,n\right)$ is the unit normal vector of plane and $p=({x^{\prime }}_{w},{y^{\prime }}_{w},{z^{\prime }}_{w})$ is the coordinates of a point on the target plane in the world coordinate system.

The two porcelain beads centers of $P_{1}$ and $P_{2}$ are obtained by CMM. Several points on the target plane are measured by CMM, and the parameters of direction vector $\vec{V}$ and fixed point p are obtained by plane fitting. The coordinates of each point in the various coordinate systems are listed in Table 2.

A central aim of calibration is to be able to obtain the coordinate of $p_{l}\left(x_{w0},y_{w0},z_{w0}\right)$ in the world coordinate system.

$p_{1}$ and $p_{2}$ are the projection onto the target plane of $P_{1}$ and $P_{2}$. The coordinates of $p_{1}$ and $p_{2}$ can be calculated by

(13) $$\left[\begin{array}{c} x_{w1}^{}\\ y_{w1}^{}\\ z_{w1}^{} \end{array}\right]=\left[\begin{array}{c} X_{w1}\\ Y_{w1}\\ Z_{w1} \end{array}\right]-d_{1}\left[\begin{array}{c} l\\ m\\ n \end{array}\right]$$

(14) $$\left[\begin{array}{c} x_{w2}^{}\\ y_{w2}^{}\\ z_{w2}^{} \end{array}\right]=\left[\begin{array}{c} X_{w2}\\ Y_{w2}\\ Z_{w2} \end{array}\right]-d_{2}\left[\begin{array}{c} l\\ m\\ n \end{array}\right]$$

where $d_{1}$ and $d_{2}$ can be calculated by

(15) $$d_{1}=\left|l(X_{w1}-x_{w}^{\prime })+m(Y_{w1}-y_{w}^{\prime })+n(Z_{w1}-z_{w}^{\prime })\right|$$

(16) $$d_{2}=\left|l(X_{w2}-x_{w}^{\prime })+m(Y_{w2}-y_{w}^{\prime })+n(Z_{w2}-z_{w}^{\prime })\right|$$

Based on the introduction of $o_{v}-x_{v}y_{v}z_{v}$ described above, the transformation from $o_{w}-x_{w}y_{w}z_{w}$ to $o_{v}-x_{v}y_{v}z_{v}$ is defined as

(17) $$\left[\begin{array}{c} x_{v}\\ y_{v}\\ z_{v}\\ 1 \end{array}\right]=\left[\begin{array}{cccc} \cos\theta +\omega_{x}^{2}(1-\cos\theta ) & \omega_{x}\omega_{y}(1-\cos\theta )-\omega_{z}\sin\theta & \omega_{y}\sin\theta +\omega_{x}\omega_{z}(1-\cos\theta ) & -x_{w1}^{\prime }\\ \omega_{z}\sin\theta +\omega_{x}\omega_{y}(1-\cos\theta ) & \cos\theta +\omega_{y}^{2}(1-\cos\theta ) & \omega_{y}\omega_{z}(1-\cos\theta )-\omega_{x}\sin\theta & -y_{w1}^{\prime }\\ \omega_{x}\omega_{z}(1-\cos\theta )-\omega_{y}\sin\theta & \omega_{x}\sin\theta +\omega_{y}\omega_{z}(1-\cos\theta ) & \cos\theta +\omega_{z}^{2}(1-\cos\theta ) & 0\\ 0 & 0 & 0 & 1 \end{array}\right]\left[\begin{array}{c} x_{w}\\ y_{w}\\ z_{w}\\ 1 \end{array}\right]=\left[\begin{array}{cc} R & T\\ 0 & 1 \end{array}\right]\left[\begin{array}{c} x_{w}\\ y_{w}\\ z_{w}\\ 1 \end{array}\right]$$

where $\vec{w}=\frac{\vec{V}\times \vec{k}}{\left|\vec{V}\times \vec{k}\right|}$ is the unit normal vector of the rotation axis, $\theta =\arccos\left(\frac{\vec{V}\cdot \vec{k}}{\left|\vec{V}\right|\left|\vec{k}\right|}\right)$ and $\vec{k}=\left(0,0,1\right)$. The coordinates of $p_{1}$ and $p_{2}$ in $o_{v}-x_{v}y_{v}z_{v}$ can be calculated by

(18) $$\left[\begin{array}{c} x_{v1}\\ y_{v1}\\ z_{v1}\\ 1 \end{array}\right]=\left[\begin{array}{cc} R & T\\ 0 & 1 \end{array}\right]\left[\begin{array}{c} {x^{\prime }}_{w1}^{}\\ {y^{\prime }}_{w1}^{}\\ {z^{\prime }}_{w1}^{}\\ 1 \end{array}\right]=\left[\begin{array}{c} 0\\ 0\\ z_{c1}\\ 1 \end{array}\right]$$

(19) $$\left[\begin{array}{c} x_{v2}\\ y_{v2}\\ z_{v2}\\ 1 \end{array}\right]=\left[\begin{array}{cc} R & T\\ 0 & 1 \end{array}\right]\left[\begin{array}{c} {x^{\prime }}_{w2}^{}\\ {y^{\prime }}_{w2}^{}\\ {z^{\prime }}_{w2}^{}\\ 1 \end{array}\right]$$

Because the $z_{v}$ axis is perpendicular to the target plane, the relationship of the $z_{v}$ axis coordinates of $p_{l}$, $p_{1}$ and $p_{2}$ is obtained by

(20) $$z_{v0}=z_{v1}=z_{v2}$$

The vector $\vec{p_{1}p_{2}}$ is expressed as

(21) $$\vec{p_{1}p_{2}}=\left(x_{v2}-x_{v1},y_{v2}-y_{v1},0\right)$$

In order to ensure that $u^{\prime }$ axis is parallel to the $x_{v}$ axis, the vector $\vec{{p^{\prime }}_{1}{p^{\prime }}_{2}}$ is expressed as

(22) $$\vec{{p^{\prime }}_{1}{p^{\prime }}_{2}}=\left(u_{2}-u_{1},v_{2}-v_{1},0\right)$$

Similarly, the transformation from o−uv to $o^{\prime }-u^{\prime }v^{\prime }$ is defined as

(23) $$\left[\begin{array}{c} u^{\prime }\\ v^{\prime }\\ 1 \end{array}\right]=\left[\begin{array}{ccc} \cos\theta^{\prime } & \sin\theta^{\prime } & -u_{1}\\ -\sin\theta^{\prime } & \cos\theta^{\prime } & -v_{1}\\ 0 & 0 & 1 \end{array}\right]\left[\begin{array}{c} u\\ v\\ 1 \end{array}\right]$$

where $\theta^{\prime }=\arccos\left(\frac{\vec{p_{1}p_{2}}\cdot \vec{{p^{\prime }}_{1}{p^{\prime }}_{2}}}{\left|\vec{p_{1}p_{2}}\right|\left|\vec{{p^{\prime }}_{1}{p^{\prime }}_{2}}\right|}\right)$. The method used to get the coordinates of ${p^{\prime }}_{1}$, ${p^{\prime }}_{2}$ and ${p^{\prime }}_{l}$ in o−uv is introduced in Section 4. The coordinates of ${p^{\prime }}_{1}$, ${p^{\prime }}_{2}$ and ${p^{\prime }}_{l}$ in $o^{\prime }-u^{\prime }v^{\prime }$ can be calculated by

(24) $$\left[\begin{array}{c} u_{1}^{\prime }\\ v_{1}^{\prime }\\ 1 \end{array}\right]=\left[\begin{array}{ccc} \cos\theta^{\prime } & \sin\theta^{\prime } & -u_{1}\\ -\sin\theta^{\prime } & \cos\theta^{\prime } & -v_{1}\\ 0 & 0 & 1 \end{array}\right]\left[\begin{array}{c} u_{1}\\ v_{1}\\ 1 \end{array}\right]=\left[\begin{array}{c} 0\\ 0\\ 1 \end{array}\right]$$

(25) $$\left[\begin{array}{c} u_{2}^{\prime }\\ v_{2}^{\prime }\\ 1 \end{array}\right]=\left[\begin{array}{ccc} \cos\theta^{\prime } & \sin\theta^{\prime } & -u_{1}\\ -\sin\theta^{\prime } & \cos\theta^{\prime } & -v_{1}\\ 0 & 0 & 1 \end{array}\right]\left[\begin{array}{c} u_{2}\\ v_{2}\\ 1 \end{array}\right]$$

(26) $$\left[\begin{array}{c} u_{0}^{\prime }\\ v_{0}^{\prime }\\ 1 \end{array}\right]=\left[\begin{array}{ccc} \cos\theta^{\prime } & \sin\theta^{\prime } & -u_{1}\\ -\sin\theta^{\prime } & \cos\theta^{\prime } & -v_{1}\\ 0 & 0 & 1 \end{array}\right]\left[\begin{array}{c} u_{0}\\ v_{0}\\ 1 \end{array}\right]$$

Because the image plane is parallel to the target plane, ${z^{\prime }}_{v0}={z^{\prime }}_{v1}={z^{\prime }}_{v2}$ is obtained. According to perspective projection, the equation is given by

(27) $$\frac{x_{v}}{z_{v}}=\frac{u^{\prime }}{z_{v0}^{\prime }}$$

(28) $$\frac{y_{v}}{z_{v}}=\frac{v^{\prime }}{{z^{\prime }}_{v0}}$$

Therefore, ${z^{\prime }}_{v0}$ can be calculated via Equations (27) and (28), and it can be submitted back to Equations (27) and (28) to calculate $x_{v0}$ and $y_{v0}$. Thus, the coordinate $\left(x_{c0},y_{c0},z_{c0}\right)$ can be obtained, and the coordinate of laser spot in $o_{w}-x_{w}y_{w}z_{w}$ can be calculated by

(29) $$\left[\begin{array}{c} x_{w0}\\ y_{w0}\\ z_{w0}\\ 1 \end{array}\right]={\left[\begin{array}{cc} R & T\\ 0 & 1 \end{array}\right]}^{-1}\left[\begin{array}{c} x_{c0}\\ y_{c0}\\ z_{c0}\\ 1 \end{array}\right]$$

## 4. Image Processing

### 4.1. Centroid Extraction

**Figure 5:**
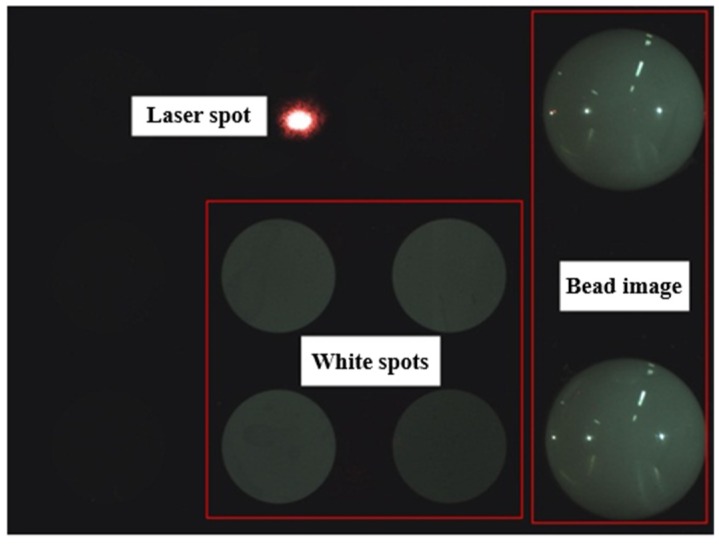
Example diagram of the laser spot and other white spots.

**Figure 6:**
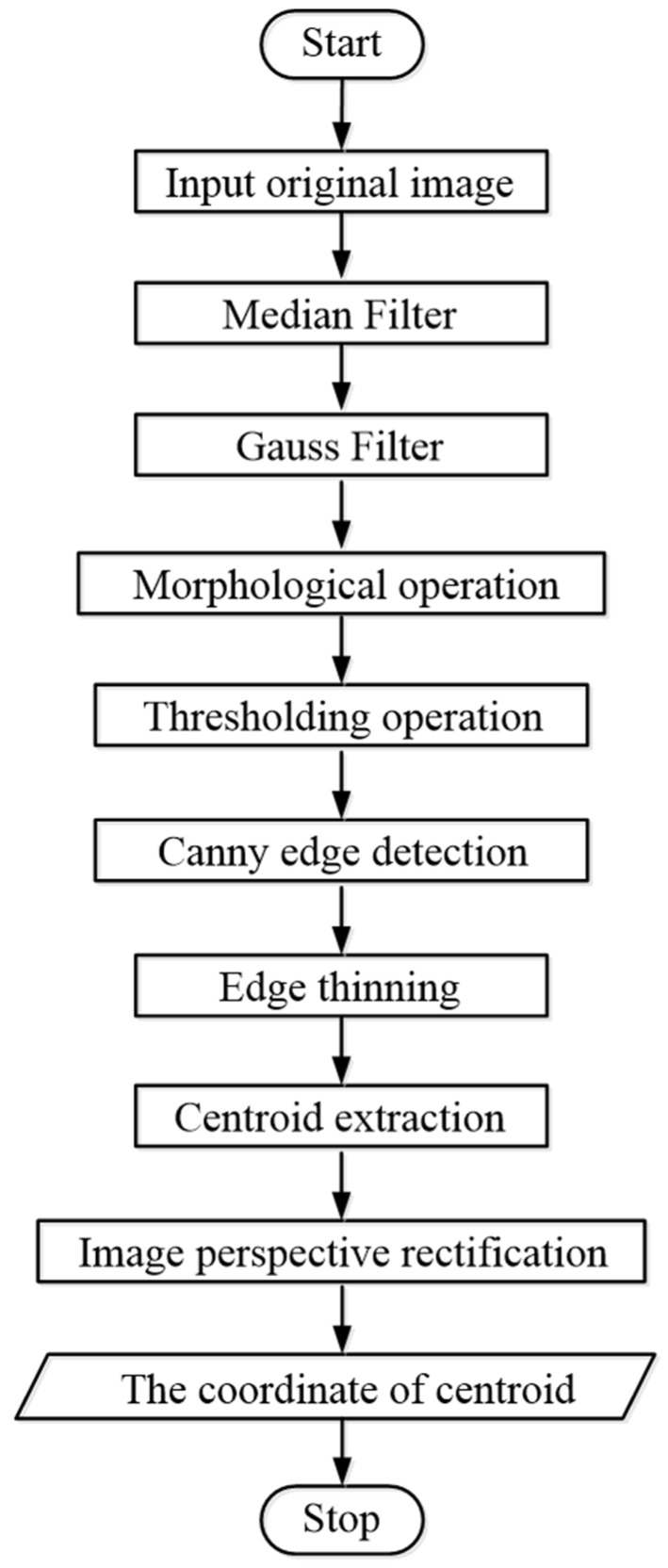
Flow chart of centroid extraction.

**Figure 7:**
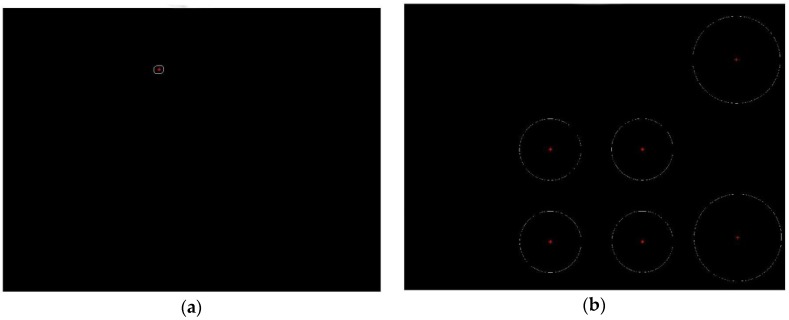
(**a**) Image of laser spot extraction; (**b**) image of white spots extraction.

One original image including one laser spot and six white spots is shown in Figure 5. The four white spots in the center of the image are used to ensure the parallelism of the target plane and the image plane, and their centers are control points. The two white spots on the right side of the image are the images of the two porcelain beads pasted to the target plane. The flow chart of centroid extraction is shown in Figure 6. The centers of the laser spot and each white spot are obtained by the centroid method [22], as shown in Figure 7.

Through the extraction method above, we can obtain the 2D coordinates of the centers of the laser spot and each white spot in o−uv. Due to the uncertainty of the camera position, the image plane is not parallel to the target plane. Therefore, it is necessary to do post-image-processing.

### 4.2. Image Perspective Rectification

**Figure 8:**
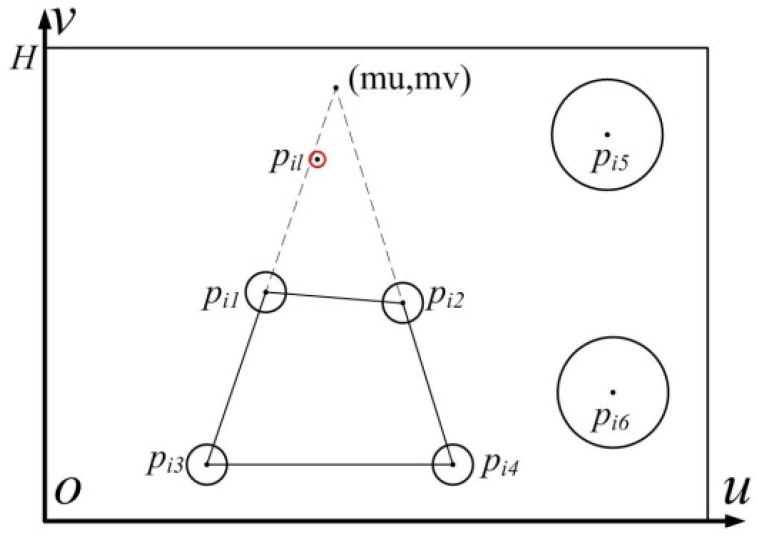
Schematic diagram of image rectification.

In order to ensure that the image plane is parallel to the target plane, the image perspective rectification method based on double vanishing point is employed [23]. The four control points are arranged in a square on the target plane. Due to the uncertainty of the camera position, they are not a square in the image. The principle diagram of image rectification is shown in Figure 8.

By analyzing the location of the four control points, the workflow of image rectification is as follows:

(1) The image is rotated to ensure that $p_{i3}p_{i4}$ is parallel to the u axis. According to the coordinates $p_{i1}$, $p_{i2}$, $p_{i3}$ and $p_{i4}$ after rotation, the vanishing point coordinate (mu,mv) is obtained.
(2) The expression of the rectification in *u*-axis direction is
  (30) $$\left\{ \begin{array}{l} v^{\prime }=v\\ u^{\prime }=u+((H-v)\times (mu-u))/(mv-v) \end{array}\right.$$
(3) where H is the width of square,
(4) and the expression of the rectification in the v axis direction is
  (31) $$\left\{ \begin{array}{l} u^{\prime \prime }=u^{\prime }\\ v^{\prime \prime }=\frac{v^{\prime }}{mu/(mu-\frac{(H-1)\times mu}{mv-v^{\prime }})} \end{array}\right.$$
(5) After the rectification in the u axis and the v axis direction, $p_{i1}p_{i3}$ is parallel to $p_{i2}p_{i4}$, but $p_{i1}p_{i2}$ is not parallel to $p_{i3}p_{i4}$. The image is rotated 90°, and the rectification in the u axis and the v axis direction is executed again.

## 5. Experiment

**Figure 9:**
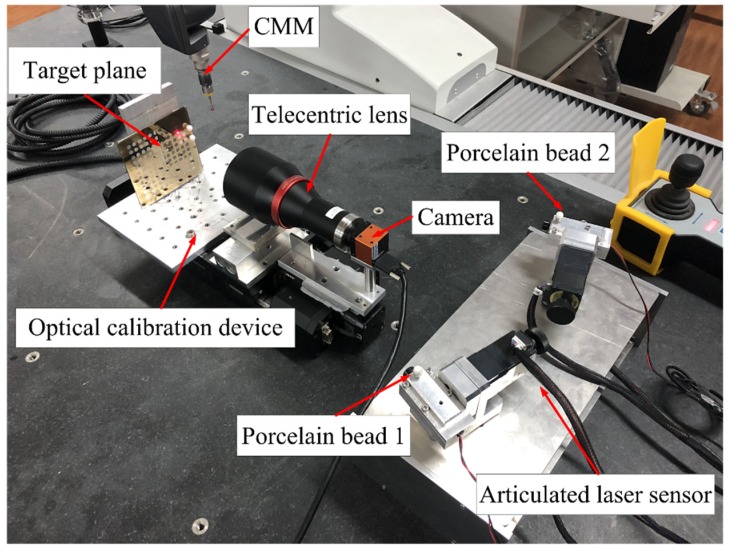
Calibration experiment diagram.

The calibration experiment site is shown in Figure 9. In the laboratory, a CMM is employed to calibrate these intrinsic and extrinsic parameters, and the CMM’s measurement coordinate system is defined as the world coordinate system.

### 5.1. Calibration of the Vertical and Horizontal Axes

As shown in Figure 9, there are two porcelain beads pasted onto the two articulated laser sensing modules, respectively. By measuring porcelain bead 1 and porcelain bead 2 rotating around the corresponding axes, the direction vectors and fixed points of the horizontal and vertical axes are obtained by plane and ellipse fitting. The intrinsic parameters under the CMM coordinate system are shown in Table 3.

### 5.2. Calibration of the Laser Beam

As shown in Figure 9, the target plane and a camera are fixed on a platform. Two porcelain beads are pasted to the target plane. The perspective projection image of a ball is usually not a standard circle, but an ellipse, and the geometric center of an ellipse is not the same as the ball-center’s real image [24]. Fortunately, a telecentric lens is employed to correct the parallax error of traditional industrial lenses.

According to the calibration principle, the calibration flow of the laser beam can be operated as follows:

(1) The articulated laser sensor is fixed on the operating platform of CMM.
(2) The optical calibration device is adjusted to ensure that the laser beam can project onto the target plane.
(3) The centers of two beads are measured by CMM, and the coordinates are recorded as $\left(X_{w1},Y_{w1},Z_{w1}\right)$ and $\left(X_{w2},Y_{w2},Z_{w2}\right)$, respectively.
(4) Nine points on the target plane are measured by CMM and used to fit a plane. The parameters of the target plane are obtained, including the unit normal vector recorded as (l,m,n) and a point on the plane recorded as $\left({x^{\prime }}_{w},{y^{\prime }}_{w},{z^{\prime }}_{w}\right)$.
(5) An image is collected by a camera with telecentric lens.
(6) Steps (2)–(5) are repeated more than seven times.
(7) The collected images are processed as described in the Section 4.
(8) Based on the least square method, a spatial line is fitted from the coordinates of the laser spots in the CMM’s coordinate system.
(9) The parameters of the laser beam are obtained, including the direction vector and a fixed point on the laser beam, as shown in Table 4.

### 5.3. Calibration of Extrinsic Parameters

The above calibration results show that the intrinsic parameters of the three axes of each articulated laser sensing module are obtained in the CMM’s coordinate system. The extrinsic parameters are received as follows

$$R_{0}=\left[\begin{array}{ccc} 0.9999 & 0.0078 & 0.0078\\ -0.0078 & 1.0000 & -0.0008\\ -0.0078 & 0.0007 & 1.0000 \end{array}\right],\;T_{0}=\left[\begin{array}{c} 60.3136\\ 0.1034\\ 10.7202 \end{array}\right]\;(\mathrm{mm}).$$

### 5.4. Verification Experiment

**Figure 10:**
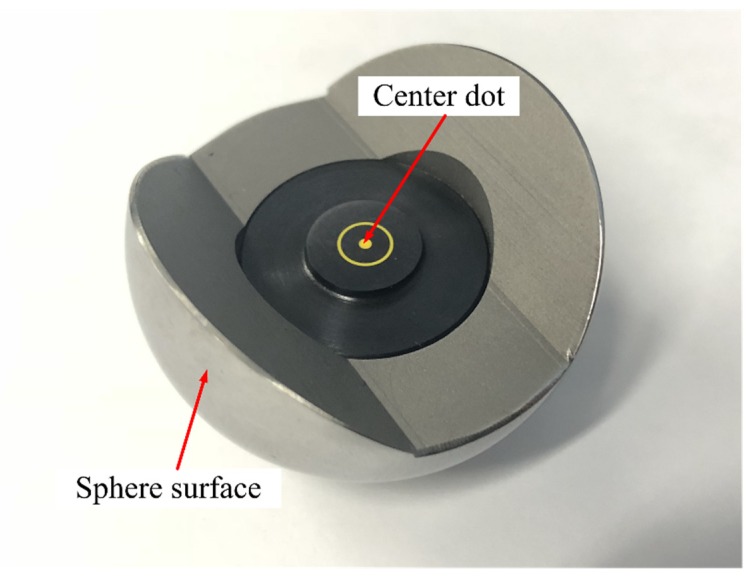
High-precision machined hemispherical target.

Further measurement experiments are needed to verify the accuracy of the proposed calibration method utilizing the intrinsic and extrinsic parameters. A high-precision machined hemispherical target with the center dot, as shown in Figure 10, is employed to achieve a high-accuracy measurement. The machining accuracy of the hemispherical target is 0.01mm.

**Figure 11:**
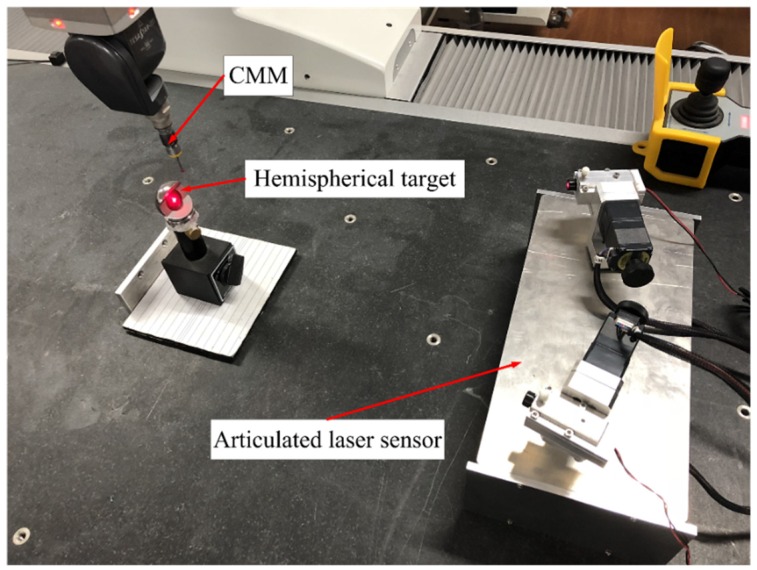
Measurement experiment diagram.

The hemispherical target is placed in different positions. The length of two positions is the measurand. The points on the sphere surface are measured by CMM, and the coordinate of center dot is obtained by sphere surface fitting, as shown in Figure 11. The intersection of the two laser beams is measured by the articulated laser sensor. The measurement results from CMM are defined as the truth values, and those from the articulated laser sensor are defined as the measured values, as shown in Table 5.

From the comparison, it is shown that the maximum distance error of the articulated laser sensor calibrated by the new method is 0.05 mm in the real experiment.

## 6. Conclusions

A novel high-accuracy calibration method of an articulated laser sensor is proposed in this paper. The system parameters to be calibrated are the spatial positions of the three axes of the articulated laser sensing module. The calibration principles of the three axes are introduced in detail. Especially, the calibration of the laser beam is elaborated, which is the key innovative aspect of the study. A novel optical calibration device is also presented to achieve high-accuracy operation, including a linear displacement guide, high-precision machined porcelain beads, and a camera with a telecentric lens. The calibration method of the laser beam consists of a perspective projection model and image processing techniques. The image processing procedure is divided into two steps: centroid extraction and image perspective rectification. The experimental results show that a maximum distance error of 0.05 mm was detected with articulated laser sensor. These encouraging results prove that this proposed calibration method is suitable for articulated laser sensors, particularly for the calibration of the laser beam.

## Figures and Tables

**Table 1:** The system parameters of the articulated laser sensor.

Category	Parameters		Physical Meaning
Intrinsic parameters	Vertical axis	Vector ${\vec{v}}_{W}$$\left(x_{WV},y_{WV},z_{WV}\right)$	Direction of vertical axis
		Point $V_{W}$$\left(x_{WVO},y_{WVO},z_{WVO}\right)$	Fixed point of vertical axis
	Horizontal axis	Vector ${\vec{h}}_{W}$$\left(x_{WH},y_{WH},z_{WH}\right)$	Direction of horizontal axis
		Point $H_{W}$$\left(x_{WHO},y_{WHO},z_{WHO}\right)$	Fixed point of horizontal axis
	Measuring axis	Vector ${\vec{p}}_{W}$$\left(x_{WM},y_{WM},z_{WM}\right)$	Direction of measuring axis
		Point $P_{W}$$\left(x_{WMO},y_{WMO},z_{WMO}\right)$	Fixed point of measuring axis
Extrinsic parameters	Rotation–translation matrix $M_{0}$	Rotation matrix $R_{0}$	Rotation from Oxyz to $O_{R}x_{R}y_{R}z_{R}$
		Translation vector $T_{0}$	Translation from Oxyz to $O_{R}x_{R}y_{R}z_{R}$

**Table 2:** Coordinates of each point in the various coordinate systems.

Point	$o_{w}-x_{w}y_{w}z_{w}$	$o_{v}-x_{v}y_{v}z_{v}$	o−uv	$o^{\prime }-u^{\prime }v^{\prime }$
$p_{l}$	$\left(x_{w0},y_{w0},z_{w0}\right)$	$\left(x_{v0,}y_{v0},z_{v0}\right)$		
$p_{l}^{\prime }$		$\left(u_{0}^{\prime },y_{v0}^{\prime },v_{0}^{\prime }\right)$	$\left(u_{0},v_{0}\right)$	$\left(u_{0}^{\prime },v_{0}^{\prime }\right)$
$P_{1}$	$\left(X_{w1},Y_{w1},Z_{w1}\right)$			
$p_{1}$	$\left(x_{w1},y_{w1},z_{w1}\right)$	$\left(x_{v1,}y_{v1},z_{v1}\right)$		
$p_{1}^{\prime }$		$\left(u_{1}^{\prime },y_{v1}^{\prime },v_{1}^{\prime }\right)$	$\left(u_{1},v_{1}\right)$	$\left(u_{1}^{\prime },v_{1}^{\prime }\right)$
$P_{1}$	$\left(X_{w2},Y_{w2},Z_{w2}\right)$			
$p_{2}$	$\left(x_{w2},y_{w2},z_{w2}\right)$	$\left(x_{v2,}y_{v2},z_{v2}\right)$		
$p_{2}^{\prime }$		$\left(u_{2}^{\prime },y_{v2}^{\prime },v_{2}^{\prime }\right)$	$\left(u_{2},v_{2}\right)$	$\left(u_{2}^{\prime },v_{2}^{\prime }\right)$

**Table 3:** Intrinsic parameters of the vertical and horizontal axes (mm).

Category		Intrinsic Parameters
Left module	Horizontal axis	Fixed point (160.919,162.305,−622.648) Direction vector (0.954,−0.300,0.005)
	Vertical axis	Fixed point (208.213,147.371,−604.198) Direction vector (0.006,−0.002,−0.999)
Right module	Horizontal axis	Fixed point (416.534,157.549,−622.284) Direction vector (0.973,0.232,−0.004)
	Vertical axis	Fixed point (368.818,146.245,−603.301) Direction vector (−0.002,−0.003,−0.999)

**Table 4:** Intrinsic parameters of the sight axis (mm).

Category		Intrinsic Parameters
Left module	Measuring axis	Fixed point (313.363,702.058,−618.860)
		Direction vector (0.270,0.963,0.005)
Right module	Measuring axis	Fixed point (252.987,691.699,−619.093)
		Direction vector (−0.2934,0.956,0.003)

**Table 5:** Comparison between the measured values and real values.

Point No.	Left/Right Module	Horizontal Angle (°)	Vertical Angle (°)	Measured Length (mm)	Real Length (mm)	Deviation (mm)
1	Left	0.000	0.000	91.747	91.752	−0.005
	Right	−15.775	−0.108			
2	Left	14.986	−0.057			
	Right	−0.052	0.012			
3	Left	33.143	−1.342	166.854	164.831	0.023
	Right	38.992	−1.258			
4	Left	15.786	−1.325			
	Right	17.184	−1.277			
5	Left	12.152	−1.475	172.134	172.141	−0.007
	Right	12.206	−1.447			
6	Left	9.647	19.920			
	Right	3.046	20.580			
7	Left	−8.503	20.021	157.470	157.467	0.003
	Right	−12.452	18.288			
8	Left	−31.538	19.282			
	Right	−29.141	15.717			
9	Left	−0.848	14.621	128.730	128.753	−0.023
	Right	4.000	14.582			
10	Left	11.500	13.578			
	Right	17.800	14.570			
11	Left	12.500	−1.744	352.831	352.781	0.050
	Right	−1.102	−1.754			
12	Left	26.000	12.595			
	Right	35.000	14.606			
